# Adiponectin Expression Protects against Angiotensin II-Mediated Inflammation and Accelerated Atherosclerosis

**DOI:** 10.1371/journal.pone.0086404

**Published:** 2014-01-22

**Authors:** Caroline M. W. van Stijn, Jason Kim, Grant D. Barish, Uwe J. F. Tietge, Rajendra K. Tangirala

**Affiliations:** 1 Division of Endocrinology, Diabetes and Hypertension, David Geffen School of Medicine at University of California Los Angeles, Los Angeles, California, United States of America; 2 Division of Endocrinology, Northwestern University Feinberg School of Medicine, Chicago, Illinois, United States of America; 3 Department of Pediatrics, University of Groningen Medical Center, Groningen, The Netherlands; University of Illinois College of Medicine, United States of America

## Abstract

Adiponectin (APN), an adipocytokine produced by adipose tissue, exerts pleiotropic actions regulating inflammation, metabolism and vascular homeostasis. APN levels are inversely correlated with obesity, type-2 diabetes, hypertension and cardiovascular disease. Although renin angiotensin system (RAS) activation in these interrelated metabolic syndrome components increases angiotensin II (AngII) levels leading to vascular damage, it is unknown whether APN under these conditions provides atheroprotection. We investigated whether increasing plasma APN provides atheroprotection in a hypertensive and accelerated atherosclerosis model. Using adenoviral gene transfer, sustained APN expression increased plasma levels of total and high-molecular weight APN, leading to a significant elevation of plasma HDL-cholesterol (HDL-C). Elevated APN levels were strongly atheroprotective, yet had no impact on blood pressure. Notably, gene expression analyses revealed that APN significantly inhibited the expression of pro-inflammatory and atherogenic genes while it increased the expression of the anti-inflammatory cytokine, IL-10 and the cholesterol efflux transporters, ABCA1 and ABCG1 in the artery wall. These findings suggest that increasing APN levels may be an effective therapeutic strategy to inhibit vascular inflammation and accelerated atherosclerosis associated with RAS activation in the metabolic syndrome.

## Introduction

Obesity is closely linked to the metabolic syndrome including dyslipidemia, insulin resistance and hypertension, which promote the development of diabetes and atherosclerotic cardiovascular disease. Adipose tissue is now recognized as an endocrine organ that produces multiple bioactive mediators, known as adipokines that play a functional role in the regulation of metabolism, inflammation and tissue remodeling [1], [2]. In obesity-related disorders, excess visceral fat accumulation leads to adipose macrophage infiltration and metabolic dysfunction contributing to low-grade local and systemic inflammation, which contribute to the pathogenesis of cardiovascular complications [3], [4].

APN (also known as apM1, AdipoQ, Acrp30, GBP-28) is an adipokine that has emerged as an important therapeutic target due to its pleiotropic actions regulating metabolism, inflammation, immune response and vascular homeostasis [5], [6]. Structurally, APN contains a collagen-domain at its N-terminus and a globular domain at the C-terminus with a sequence homology to complement C1q [7], [8], [9]. APN circulates at relatively high concentrations in plasma (3–30 µg/mL) and forms three high-order oligomeric forms: a high molecular weight form (HMW, 12–32-mer), a medium molecular weight form (MMW, hexamer) and a low molecular weight form (LMW, trimer) [9], [10], [11]. In contrast to other adipokines, adipose expression and plasma concentrations of APN are reduced in the metabolic syndrome, diabetes and cardiovascular disease [6], [12], [13]. Indeed, clinical studies have demonstrated that plasma APN levels negatively correlate with visceral adiposity, hyperlipidemia, and insulin resistance, but correlate positively with HDL-C levels [14], [15]. Furthermore, low plasma HMW APN levels rather than total APN levels are a superior biomarker of insulin resistance and metabolic syndrome [10], [11]. Notably, APN mediates its effects through interaction with specific APN receptors, AdipoR1 and AdipoR2, to activate signal transduction pathways mediated by AMPK and PPARα in target tissues [13] ^,^ [16]. Thus, agents that stimulate the expression of APN or APN receptor agonists are promising therapeutic targets for the metabolic syndrome.

The RAS is a key regulator of blood pressure and cardiovascular function [17]. RAS activation in the metabolic syndrome leads to increased AngII levels, driving arterial wall inflammation, oxidative stress and atherosclerotic lesion development [18], [19], [20]. Studies in high-fructose fed rats have shown that RAS activation reduces plasma APN levels due to increased oxidative stress which inhibits adipokine production [21], [22]. Clinical studies have not only shown a relationship between plasma APN levels and hypertension but also that low APN levels are a predictor of future hypertension [23], [24], [25]. Furthermore, APN-deficient mice under high-salt stress develop hypertension that can be ameliorated by APN expression. Similarly, APN expression decreases hypertension in obese and hypertensive KKAy mice [26]. Although RAS inhibition is known to provide cardiovascular protection, it produces only modest improvements in vascular inflammation and the beneficial effects are mostly independent of blood pressure reduction [17]. Intriguingly, RAS inhibition by either angiotensin converting enzyme (ACE) inhibitors or angiotensin receptor (AT1) blockers was associated with increased circulating APN levels and improved cardiovascular status [27], [28], [29].

APN exerts potent anti-inflammatory and anti-atherogenic effects in vascular cells [12], [30], thus, linking adipose tissue physiology to vascular function. Available *in vitro* evidence suggests that APN protects against AngII and TNFα-induced endothelial dysfunction and decreases the expression of endothelial adhesion molecules [31], [32], [33]. APN also inhibits the expression of scavenger receptor, SRA1 and upregulates expression of the cholesterol efflux promoting transporter, ABCA1, in monocyte-derived macrophages [34]. In spite of these effects *in vitro* and *in vivo,* the atheroprotective role of APN in dietary models of atherosclerosis has remained unclear. Studies using either the loss- or gain-of-APN function in mouse models have shown a protective role for APN against endothelial dysfunction and hypertension [26], [35]. Although short-term adenoviral APN expression inhibited atherosclerosis [36], subsequent studies by Nawrocki *et al.* found that neither APN-deficiency nor APN overexpression affected atherosclerosis in LDLR^−/−^ mice fed a low-fat or high-fat diet as well as in APN-deficient ApoE mice [37]. However, no studies have assessed the impact of APN in model of metabolic syndrome with cardiovascular complications, especially vascular inflammation and hypertension.

In the metabolic syndrome, RAS activation contributes to vascular damage, inflammation and atherosclerosis. It remains to be determined whether APN expression under these conditions provides atheroprotection. Here, we addressed the hypothesis that increasing plasma APN levels provides therapeutic benefit by inhibiting AngII-mediated vascular inflammation and atherosclerotic lesion development in a hypertensive and accelerated atherosclerosis LDLR^−/−^ model [38]. Using this model, we demonstrate that APN exerts profound anti-inflammatory and anti-atherogenic effects, without blood pressure reduction, leading to atheroprotection.

## Methods

### Animal Studies

Male LDLR^−/−^ mice (3 months of age) from Jackson Laboratories (Bar Harbor, ME, USA) were randomly divided into 3 groups (n = 12–16 mice/group) and fed a high-fat (HF) diet (Research Diets, New Brunswick, NJ, USA, Western diet D12079B; protein 17 kcal%; carbohydrate 43 kcal%; fat 41 kcal%). Mice were administered either vehicle (PBS), GFP virus (2×10^8^ PFU) or APN virus (2×10^8^ PFU) via retro-orbital injection. One day after injection the mice were infused with AngII (Calbiochem, Gibbstown, NJ, USA) or PBS through subcutaneous implantation of osmotic mini-pumps (Alzet, Cupertino, CA, USA) containing AngII (2.5 µg/kg per minute) in PBS or PBS alone for 4 or 8 weeks. Blood pressures were measured daily until plateau level was attained and then weekly for the duration of the study by the tail-cuff method using BP-200 form Visitech system Inc. (Apex, NC, USA). All animals used in this study were approved by the Animal Research Committee, University of California, Los Angeles, CA.

### Quantification of Atherosclerosis and Immunohistochemistry

Mice were euthanized and perfused with 7.5% sucrose in 4% paraformaldehyde. 1 mM EDTA, pH 7.4. The entire aorta was dissected out, split longitudinally, pinned flat in a dissociation pan, and stained with Sudan IV to determine lesion area. Images were captured by Sony 3-CCD video camera and analyzed using ImagePro image analyzing software (Media Cybernatic Inc., Bethesda, MD, USA). The extent of atherosclerotic lesion formation is expressed as the percentage of the total aortic surface area covered by lesions [39]. The total lesion area in Oil Red O-stained aortic root sections was determined on digitized images from 3 equally spaced sections per mouse (n = 5 per group). Immunohistochemical analyses of aortic root sections were performed as described [40] with antibodies specific for macrophages MOMA2 and APN. Collagen in lesions was detected by using Masson trichrome stain. The macrophage and collagen-positive areas in stained aortic root sections (5 equally spaced sections per mouse n = 3–5 per group) were quantified using Image Pro Plus image analysis and the data are expressed as percent of total lesion area.

### Construction of Recombinant APN Adenovirus

The mouse APN cDNA was inserted in the pAxCAwT plasmid (TAKARA Biomedical, Shiga, Japan) to generate pAxCAwt-mouse APN. The resulting plasmid containing APN cDNA was under the control of CAG promoter (CMW enhancer, chicken β-actin promoter, and part of an untranslated region of rabbit β-globin). The virus was expanded by ViraQuest, Inc. (North Liberty, IA, USA).

### Analysis of Gene Expression

Gene expression in whole aortas was measured by quantitative real-time PCR [38]. For RNA isolation, aortas from mice prepared as described were perfused with ice-cold PBS and quickly frozen in liquid nitrogen and stored at −80°C until RNA extraction. Total RNA from the aortas was isolated using Rneasy kit and DNase (QIAGEN, Valencia, CA, USA), then reverse transcribed into cDNA using Taqman Reverse Transcription Reagent Kit (Applied Biosystems, Foster City, CA, USA). Quantitative real-time PCR (SYBR green) was performed with an ABI-PRISM 7500 system (Applied Biosystems) in a total volume of 20 µl, using a TaqMan PCR Core Reagent Kit (Applied Biosystems). The reaction profile was 2 min 50°C, 10 min 95°C and 40 cycles of 15 min 95°C and 1 min 60°C followed by 15 sec 95°C, 1 min 60°C and 15 sec 95°C. Each sample was analyzed in duplicate and normalized to the value of GAPDH mRNA expression. The primer sequences for investigated genes are shown in Table-1.

**Table 1:** Primer sequences of genes used for quantification of mRNA levels by real-time PCR.

Gene Product	Forward and reverse primers (5′→3′)
CD68	ABI: Mm00839636_g1
AdipoR1	ABI: Mm01291331_m1
AdipoR2	Mm01184030_m
ICAM	ABI: Mm00516023_m1
MCP-1	Forward TTA AAA ACC TGG ATC GGA ACC AAReverse GCA TTA GCT TCA GAT TTA CGG GT
CCR2	Forward ATC CAC GGC ATA CTA TCA ACA TCReverse CAA GGC TCA CCA TCA TCG TAG
TNF-α	Forward CCC TCA CAC TCA GAT CAT CTT CTReverse GCT ACG ACG TGG GCT ACA G
CD11c	Forward AGA CGT GCC AGT CAG CAT CAA CReverse CTA TTC CGA TAG CAT TGG GTG AGT G
IL-6	Forward GGT GCC CTG CCA GTA TTC TCReverse GGC TCC CAA CAC AGG ATG A
IL-10	Forward GCT CTT ACT GAC TGG CAT GAGReverse CGC AGC TCT AGG AGC ATG TG
IL-12p40	Forward TGG TTT GCC ATC GTT TTG CTGReverse ACA GGT GAG GTT CAC TGT TTC
Osteopontin	Forward AGC AAG AAA CTC TTC CAA GCA AReverse GTG AGA TTC GTC AGA TTC ATC CG
SR-A1	Forward GCA CAA TCT GTG ATG ATC GCTReverse CCC AGC ATC TTC TGA ATG TGA A
SR-B1	Forward TTT GGA GTG GTA GTA AAA AGG GCReverse TGA CAT CAG GGA CTC AGA GTA G
CD36	Forward AGA AGG CGG TAG ACC AGA CReverse GTA GGG GGA TTT CTC CTT GGA
ABCA1	Forward AAA ACC GCA GAC ATC CTT CAGReverse CAT ACC GAA ACT CGT TCA CCC
ABCG1	Forward CTT TCC TAC TCT GTA CCC GAG GReverse CGG GGC ATT CCA TTG ATA AGG
AT1R	Forward AAC AGC TTG GTG GTG ATC GTCReverse CAT AGC GGT ATA GAC AGC CCA
AT2R	Forward ATG CTT GGG GCA ACT TCA CTAReverse GCA GCA AGA GAA GGG CTT CA
ApoAI	Forward GGC ACG TAT GGC AGC AAG ATReverse CCA AGG AGG AGG ATT CAA ACT G
ApoAII	Forward TGG TCG CAC TGC TGG TAA CReverse TTT GCC ATA TTC AGT CAT GCT CT
ApoB100	Forward TTG GCA AAC TGC ATA GCA TCCReverse TCA AAT TGG GAC TCT CCT TTA GC
PPARα	Forward AGA GCC CCA TCT GTC CTC TCReverse ACT GGT AGT CTG CAA AAC CAA A

### Western Blot Analyses

For the detection of ApoA1, ApoB and APN in plasma and liver extracts from mice were resuspended in 40 µL of Laemli buffer (Bio-Rad) and heated at 95°C for 5 min. The samples were subjected to 10–20% linear gradient SDS-polyacrylamide gel electrophoresis and transferred to nitrocellulose membranes. The presence of ApoA1, ApoB and APN was detected using anti-mouse ApoA1, ApoB and APN antibodies as primary and horseradish peroxidase-labeled rabbit anti-muse IgG as secondary antibody.

### Determination of Plasma Lipid Levels and Metabolic Factors

Plasma samples from mice were collected after fasting and total cholesterol, HDL-C, triglycerides and free fatty acids were analyzed by enzymatic methods (Wako Chemicals USA, Inc., Richmond, VA, USA). Blood glucose levels were measured by one-touch glucose monitoring system (Wako). Serum APN levels were measured by Adiponectin ELISA kit (Otsuka Pharmaceuticals Inc., Rockville, MD, USA). The distribution of cholesterol in the lipoprotein subclasses in plasma was determined by Fast performance liquid chromatography (FPLC) of 200 µL of pooled plasma samples from each of the experimental groups using a Superose 6 column (LKB Biotechnology, Uppsala, Sweden). Total cholesterol content in the lipoprotein fractions was determined using enzymatic colorimetric assays (see above).

### Determination of Plasma APN Oligomeric Form Levels by Western Blot

To determine the amount of APN in plasma samples of mice and the oligomeric forms of APN in the serum, 0.1 µl of serum was loaded onto a 4–12% NuPAGE gel (Invitrogen). The gel for the APN oligomeric forms was run under non-reducing, non-denaturing conditions according to previously published method [10], [11]. The gels were blotted onto nitrocellulose with the iBlot dry blotting system (Invitrogen). APN was visualized using anti-mouse APN antibody (R&D systems, Minneapolis, MN, USA).

### Statistical Analyses

All data were expressed as mean ± standard deviation. Statistical significance was determined by 1-way ANOVA, Newman-Keuls post-test and unpaired Student *t*-test with GraphPad Prism software. Experimental groups were compared to the vehicle group with the Dunnet test. Statistical differences for all comparisons was set at p<0.05.

## Results

### Long-term APN Expression Alters the Distribution of APN Oligomeric Forms in an AngII-induced Hypertensive Model of Accelerated Atherosclerosis

**Figure 1:**
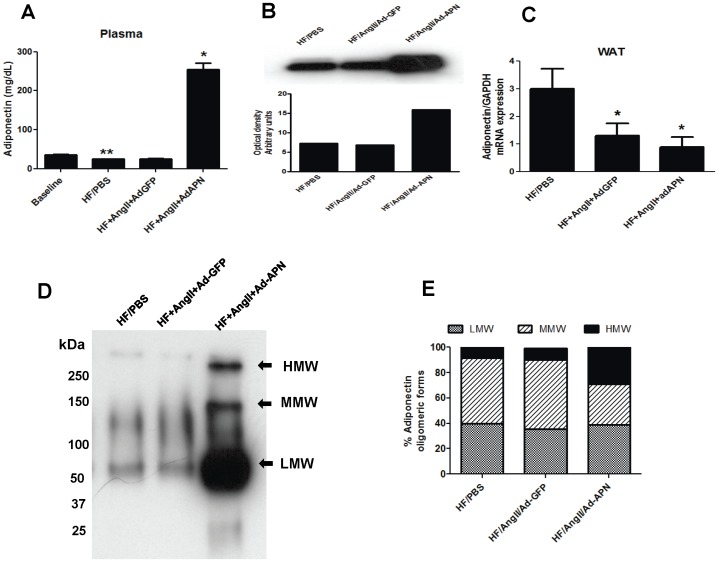
Effect of adenoviral APN expression on plasma levels, endogenous adipose tissue APN expression and distribution of plasma APN oligomeric forms in AngII-infused LDLR^−/−^ mice. **A:** Plasma APN levels in AdGFP and AdAPN-injected LDLR^−/−^ mice, infused with PBS or AngII and fed high-fat diet for 8 weeks, were measured by ELISA (**p<0.05 baseline vs HF/PBS; *p<0.001 vs HF/AngII/AdAPN, n = 8/group) and **B:** by Western blotting. **C:** APN mRNA levels in white adipose tissue were analyzed QRT-PCR and normalized to GAPDH (*p<0.05 vs HF/PBS, n = 5/group). **D:** APN isoform distribution in plasma from HF/PBS, HF/AngII/AdGFP and HF/AngII/AdAPN LDLR^−/−^ mice was resolved by non-denaturing Western blot analysis (LMW = low molecular weight, MMW = medium molecular weight, HMW = high molecular weight form). **E:** Plasma APN oligomeric forms quantified were expressed as percent of total APN levels (*p<0.01, HF/AngII/AdGFP vs. HF/AngII/AdAPN, n = 3/group).

To investigate the effects of long-term APN expression on AngII-mediated vascular inflammation and atherosclerosis, we used hepatic adenoviral gene transfer to achieve chronic elevation of plasma APN levels in the AngII-induced LDLR^−/−^ model of atherosclerosis. At baseline, plasma APN levels were 30.4 µg/mL in chow diet-fed LDLR^−/−^ mice. After 8 weeks of high-fat (HF) feeding, the plasma APN levels were significantly reduced compared to baseline APN levels (38% reduction)(Figure 1A). To induce APN expression and increase circulating APN levels, AngII-infused male LDLR^−/−^ mice fed HF were intravenously injected with adenovirus expressing mouse APN (AdAPN) or control adenovirus expressing green florescent protein (AdGFP). Determination of plasma APN levels by ELISA after viral injection revealed peak APN levels at day 7 (17-fold elevation vs GFP). The APN levels on day 56 after viral injection demonstrated a 10-fold elevation in AdAPN mice compared those in Ad-GFP mice (Figure 1A). Consistent with these results, Western blot analysis of plasma samples at 8 weeks of APN expression showed more than 5-fold increase in APN in AdAPN mice compared with AdGFP mice (Figure 1B). Next, we determined the effects of AngII infusion and exogenous APN on adipose APN expression. Analysis of adipose mRNA levels after 8 weeks of treatment showed that AngII infusion significantly suppressed APN levels in the adipose tissue which was unaffected adenoviral expression of APN in the liver (Figure 1C).

**Figure 2:**
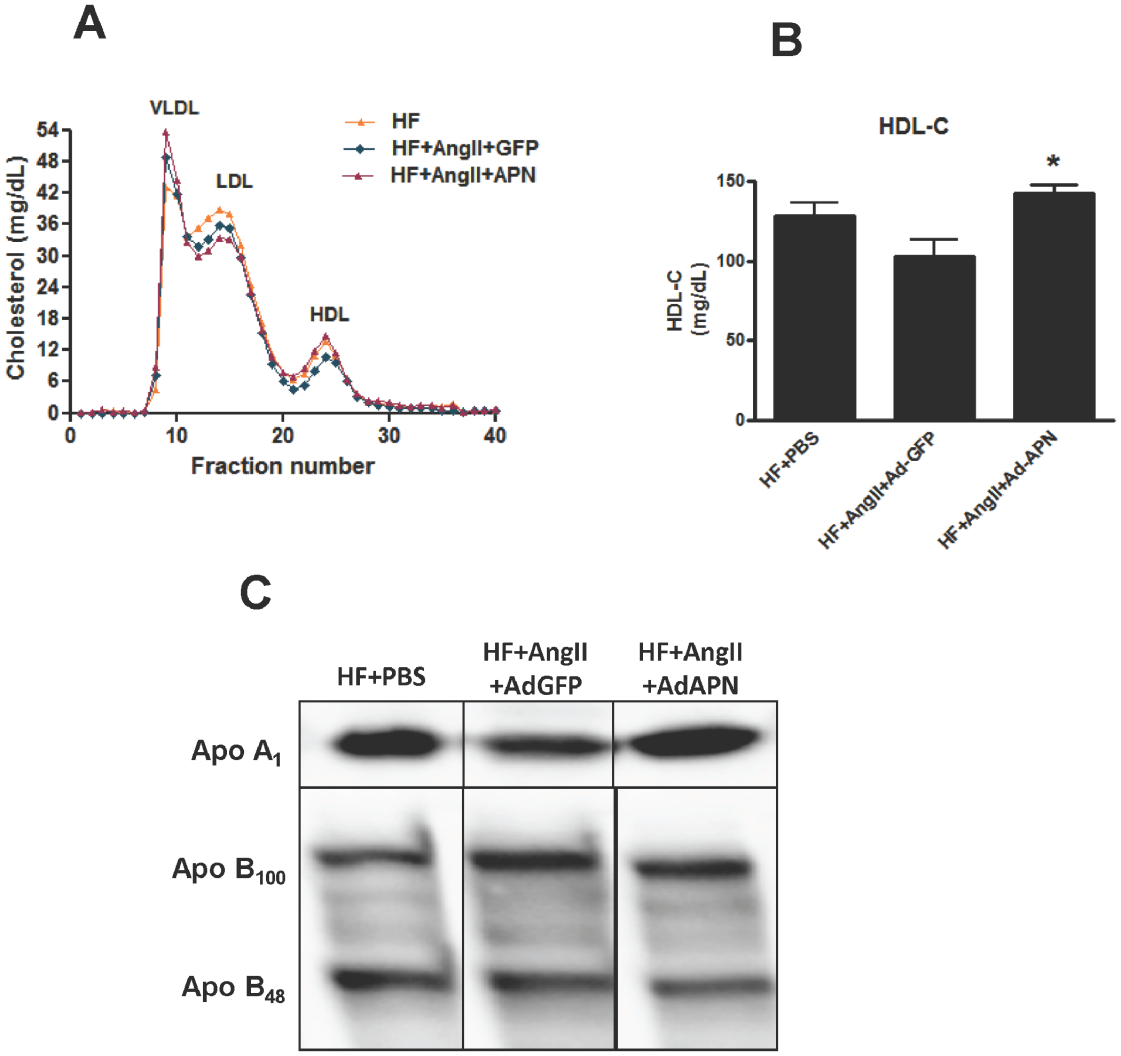
Effect of elevated adiponectin levels on plasma lipoprotein profile, HDL-cholesterol and apolipoprotein levels. **A:** Plasma lipoprotein profiles of HF/PBS, HF/AngII/AdGFP and HF/AngII/AdAPN pooled samples were determined by fast performance liquid chromatography. **B:** Plasma HDL-C levels determined by enzymatic method (*p<0.001 HF/AngII/AdGFP vs HF/AngII/AdAPN, n = 8/group ). (**C**) Western blot analysis of plasma apolipoproteins A1, B_100_ and B_48_ levels in pooled samples from HF/PBS, HF/AngII/AdGFP and HF/AngII/AdAPN after 8 weeks of adiponectin expression.

Plasma APN circulates in three major oligomeric forms: HMW, MMW and LMW forms [10], [11] thus we next determined the effect of adenoviral APN expression on the distribution of APN oligomeric forms in the AngII-induced model of atherosclerosis. In plasma obtained after 8 weeks of treatment, measurement of APN using non-reducing and non-denaturing gel electrophoresis revealed that APN expression in AdAPN mice significantly increased the proportion of HMW APN (Figure 2D). Correspondingly, there was a reduction in the proportion of MMW APN in AdAPN mice compared to AdGFP controls (Figure 1E). A comparison of APN oligomeric forms between HF/AngII/AdGFP mice and HF/PBS mice revealed that AngII treatment had no significant effect on APN distribution (Figure 1E). These results indicate that more than 30% of plasma APN circulates in the HMW form in AdAPN mice. Since HMW APN is a known to improve component of the metabolic syndrome, AdAPN mice are thus a model for APN therapy.

### APN Expression Improves HDL-C Levels and Hepatic Apolipoprotein Expression without Blood Pressure Reduction

AngII infusion in LDLR^−/−^ mice fed high-fat diet for 8 weeks in Ad-GFP and Ad-APN mice significantly increased their blood pressure (>40 mmHg. P<0.01) compared to control mice infused with PBS (Table-2). APN expression had no significant effect on AngII-induced blood pressure (Table-2). Both body and adipose tissue weights were significantly reduced in AngII-infused AdGFP and AdAPN mice compared with control PBS-infused mice (Table-2). Notably, the reduction of liver weight due to AngII-infusion after 8 weeks was absent in AdAPN mice (Table-2). Plasma total cholesterol, triglycerides, fatty acids, and fasting glucose levels at 8 weeks of treatment showed no significant differences between AdGFP and AdAPN mice (Table-2).

**Table 2:** Characteristics of high-fat (HF)-fed LDLR^−/−^ mice infused with AngII or PBS expressing AdGFP or AdAPN.

	HF/PBS (n = 12)	HF/AngII/AdGFP (n = 16)	HF/AngII/AdAPN(n = 16)
Initial BP (mm Hg)	118.5±11.0	116.4±9.4	117.7±7.6
Final BP (mm Hg)	118.0±10.5	171.8±15.0*	167.0±17.0*
Body weight (g)	32.3±2.8	21.2±7.8*	23.5±1.3*
Liver weight (g)	1.33±0.2	0.87±0.3**	1.29±0.3
Adipose tissue weight (g)	1.34±0.5	0.36±0.14	0.31±0.1
Total cholesterol (mg/dL)	837.5±185.2	825.1±69.7	875.7±206.1
Triglycerides (mg/dL)	48.0±17.0	162.0±36.6	153.0±28.9
Fatty acids (mg/dL)	42.6±5.7	47.2±2.9	49.2±6.4
Glucose (mg/dL)	396.9±51.1	288.7±53.4	320.9±41.0

* p<0.05 vs HF/PBS, **p<0.05 vs HF/AngII/AdAPN, BP = blood pressure.

A number of studies reported a positive correlation between plasma APN and HDL-C levels. Thus, we determined the impact of APN expression on plasma lipoproteins by analyzing lipoprotein profiles, HDL-C and apolipoprotein levels. APN expression significantly increased plasma HDL-C levels by 28% in AdAPN mice (Figure 2B). This increase in HDL-C was observed 7-days after adenoviral APN injection (data not shown). The effect of APN expression on plasma lipoprotein profiles was examined by measuring cholesterol distribution in lipoprotein fractions. The overall, the lipoprotein profile of Ad APN mice indicated higher HDL-C, while ApoB-containing lipoproteins appeared comparable to AdGFP controls (Figure 2A). To determine whether APN expression affects apolipoproteins associated with HDL and LDL in the plasma, we performed Western blot analyses of plasma samples obtained at 8 weeks of AngII-infusion and APN treatment. The results revealed higher plasma ApoA1 levels in AdAPN mice compared with those in AdGFP mice (Figure 2C). However, no apparent changes in plasma ApoB100 and ApoB48 protein levels were detected (Figure 2C). These data suggest that APN expression increases plasma ApoA1, the major component of HDL, which is critical for HDL-biogenesis and reverse cholesterol transport.

### Adiponectin Increases Hepatic Adiponectin Receptor and Apolipoprotein Gene Expression

**Figure 3:**
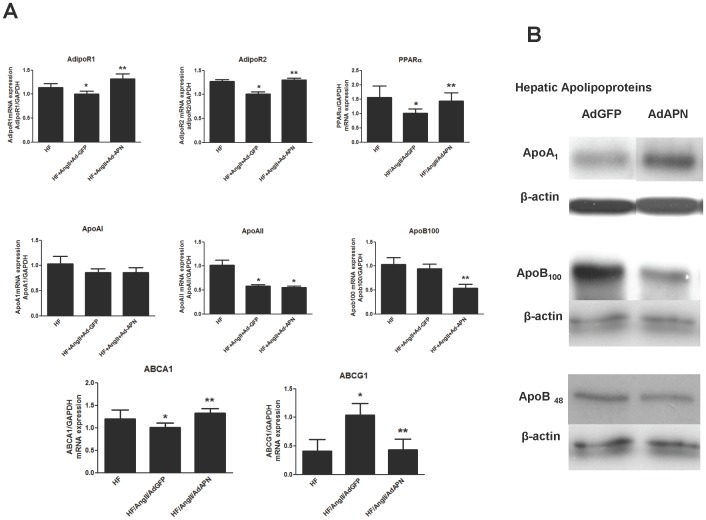
Adiponectin regulates hepatic adiponectin receptor and metabolic gene expression. **A:** RNA isolated from liver samples were analyzed for adiponectin receptor and metabolic target genes by QRT-PCR and the expression was normalized to GAPDH (*p<0.05 vs. HF, **p<0.05 vs HF/Ang/AdGFP, ANOVA, Newman-Keuls, n = 5/group). **B:** Western blot analyses of hepatic expression of apolipoproteins, ApoA_1_, ApoB_100_ and ApoB_48_. Representative Western blots were shown from the analyses performed using protein extracts derived from livers of AdGFP and AdAPN mice.

Since the liver is the central organ controlling lipid metabolism and reverse cholesterol transport [41], [42], we investigated the impact of APN on hepatic expression of APN receptors and genes regulating HDL and lipid homeostasis. Analysis of APN receptors in the liver revealed that AngII significantly suppressed the expression of AdipoR1 and AdipoR2 (p<0.05, HF/PBS vs HF/AngII/AdGFP) (Figure 3A). Interestingly, APN not only inhibited the suppressive effect of AngII, but also significantly enhanced the hepatic expression of AdipoR1 and AdipoR2 beyond the levels observed in HF/PBS mice (Figure 3A). Since APN signaling via AdipoR2 activates peroxisome proliferator-activated receptor alpha (PPARα) [13], a key gene in HDL and triglyceride metabolism, we measured PPARα mRNA expression in the liver. Consistent with AdipoR2 suppression, hepatic PPARα mRNA levels were also reduced in HF/AngII/AdGFP mice compared to those in HF/PBS mice (Figure 3A). Notably, APN mitigated the AngII suppression of hepatic PPARα expression (Figure 3A). We also sought evidence for APN regulation of apolipoprotein genes involved in lipid metabolism *in vivo* by measuring apolipoprotein, ApoA1, ApoB mRNA and protein levels. Although hepatic ApoA1 mRNA levels revealed no change after APN expression (Figure 3A), we found a substantial increase in hepatic ApoA1 protein expression by Western blot analysis (Figure 3B). Furthermore, APN significantly suppressed hepatic ApoB mRNA and ApoB_100_ protein levels and modestly reduced ApoB48 protein levels in AdAPN mice compared to AdGFP controls (Figure 3A and B).

In hepatocytes, ABCA1 plays an important role in HDL formation. Available *in vitro* studies indicate that APN increases the expression of ABCA1 but not ABCG1 in hepatocytes [43]. In this study, we provide *in vivo* evidence that APN expression significantly increased ABCA1 while it suppressed ABCG1 in the liver (AdGFP vs AdAPN, p<0.05) (Figure 3A). Furthermore, we observed increased hepatic PPARα in AdAPN mice, which is a downstream target of APN that may contribute to ABCA1 induction. These *in vivo* results indicate that APN may contribute to HDL elevation in part by increasing the expression of ABCA1 in the liver.

### Adiponectin Expression Provides Atheroprotection in a Hypertensive Model of Accelerated Atherosclerosis without Reducing Blood Pressure

**Figure 4:**
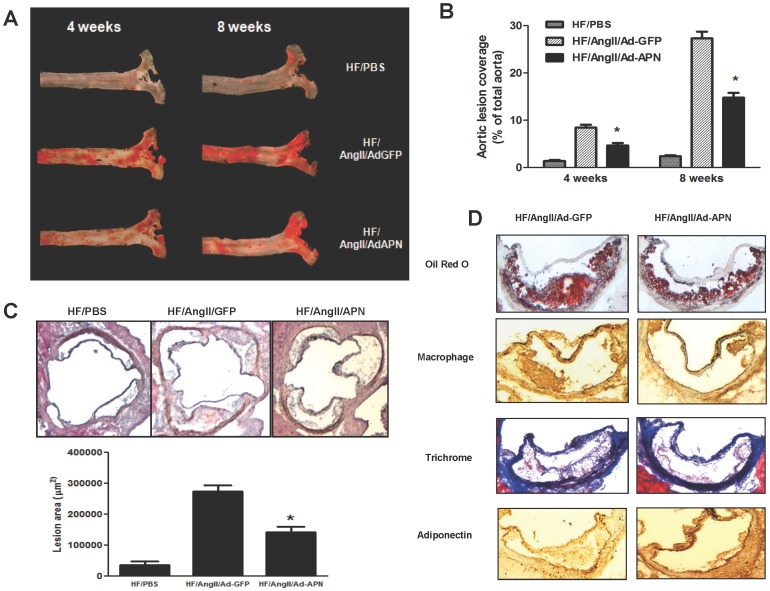
APN expression inhibits AngII-accelerated atherosclerosis and affects atherosclerotic lesion composition. **A:** Representative Sudan IV staining of *en face* aortic preparations showing aortic atherosclerotic lesions (Red) after 4 and 8 weeks of APN expression in HF/AngII/AdGFP and HF/AngII/AdAPN mice. **B:** *En face* quantification of atherosclerotic lesion areas in the aortas of HF/Ang II/AdGFP and HF/Ang II/AdAPN mice after 8 weeks of APN expression (*p<0.01, HF/AngII/AdGFP vs HF/AngII/AdAPN, n = 12–16/group). **C:** Atherosclerotic lesions in the aortic root, representative aortic sections from HF/PBS, HF/AngII/AdGFP and HF/Ang II/AdAPN groups and quantification of aortic root lesion area (*p<0.05, HF/AngII/AdGFP vs HF/AngII/AdAPN, n = 5–7/group). D. Representative aortic root sections stained for lipid (Oil Red O), macrophages (red) using MOMA2 antibody, Masson trichrome staining of lesions for collagen content (blue) and immunolocalization of APN in atherosclerotic lesions (brown) of HF/AngII/AdGFP and HF/AngII/AdAPN mice (Magnification 200 X).

We investigated whether APN provides atheroprotection against AngII-mediated inflammation and atherosclerosis using a hypertensive and accelerated atherosclerosis LDLR^−/−^ model. Previous studies in our laboratory have extensively characterized this model for a number of anti-atherogenic interventions relevant to cardiovascular complications of the metabolic syndrome in which RAS activation and AngII elevation play a pathogenic role [38], [44]. Male LDLR^−/−^ mice fed high-fat diet and infused with AngII were injected with either AdGFP or AdAPN. To determine whether APN provides atheroprotection against AngII-accelerated atherosclerosis, aortas were prepared from separate cohorts of AdGFP and AdAPN mice (n = 12–16 mice/group) after 4 or 8 weeks of treatment. The extent of atherosclerotic lesion development in entire aortas was quantified by *en face* analysis [39]. Sudan-IV stained *en face* aortic preparations after 4 and 8 weeks revealed extensive atherosclerotic lesion development throughout the aortic surface in AdGFP mice which was obviously reduced in the AdAPN mice (Figure 4A). *En face* quantification of atherosclerosis in the entire aortas after 8 weeks of treatment demonstrated that APN expression significantly inhibited AngII-accelerated atherosclerosis (48% reduction, p<0.01). (Figure 4B). In addition, quantification of lesion size in aortic root sections revealed a significant reduction of lesion size in AdAPN mice compared to that in AdGFP mice (45% reduction, p<0.05) (Figure 4C). These results provide important evidence that APN expression markedly inhibited AngII-mediated atherosclerotic lesion development in a hypertensive and accelerated atherosclerosis model.

### Adiponectin Expression Alters Atherosclerotic Lesion Composition

We examined the effect of APN expression on atherosclerotic lesion composition in mice after 8 weeks of treatment. Immunostaining of aortic root lesions with macrophage-specific antibody, MOMA2 showed a significant reduction in macrophage-positive aortic lesion area in AdAPN mice (48.3±10.3) compared with those in AdGFP (26.5±8.7) (45% reduction, p<0.05) (Figure 4C, MOMA2). Trichrome staining of aortic root lesions to detect collagen revealed significantly more collagen-positive (blue) staining, especially in the intimal layer of aortic lesions consistent with a more stable lesion phenotype in AdAPN mice (49.1±4.0)than in AdGFP mice (56.0±3.0) (12.3% increase, p<0.05) (Figure 4C, trichrome). We also examined whether APN expression contributes to increased APN localization in aortic lesions. Aortic root sections stained with anti-APN antibody revealed qualitatively strong APN-positive staining in lesions of AdAPN mice whereas only weak APN staining was detected in AdGFP lesions (Figure 4C, adiponectin).

### Adiponectin Inhibits Macrophage Recruitment and AngII-mediated Inflammation in the Artery Wall

**Figure 5:**
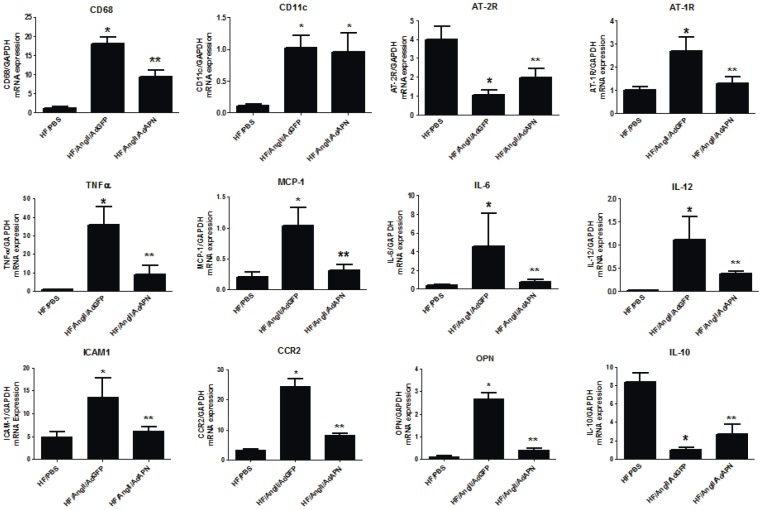
Adiponectin inhibits aortic macrophage infiltration and inflammatory gene expression and increases anti-inflammatory cytokine IL-10 expression. RNA samples from derived from aorta were analyzed for CD68, CD11c, AT-1 receptor, AT-2 receptor, MCP-1, CCR2, TNFα, IL-10, IL-12, IL-6, ICAM-1, osteopontin, gene expression by QRT-PCR and normalized to GAPDH expression. (*p<0.05 vs HF, **p<0.05 vs HF/AngII/AdGFP, ANOVA, Newman-Keuls, n = 5–7/group).

AngII is a strong inducer of macrophage recruitment, foam cell formation and inflammatory gene expression in the artery wall [38], [44]. To characterize APN-regulation of vascular inflammatory gene expression *in vivo*, we performed quantitative RT-PCR to determine the gene expression profile of aortas from AngII-infused mice. As we have shown in previous studies, AngII markedly increased the aortic expression of the CD68 macrophage marker in AdGFP mice, and interestingly, consistent with immunohistochemistry results, APN expression significantly inhibited CD68 expression (50% reduction) (AdGFP vs AdAPN, p<0.001). Similarly, a dendritic cell marker, CD11C, also increased significantly in aortas after AngII-infusion, yet APN expression did not affect CD11C levels in the artery wall (Figure 5). Analysis of AngII receptors in the aorta revealed that AngII increased AT1R while it suppressed AT2R expression (Figure 5). Interestingly, APN expression counteracted AngII effects on AngII receptor expression by suppressing AT1R while enhancing AT2R expression in the artery wall (Figure 5). These results indicate that APN modulates aortic AngII receptor expression to inhibit AngII-mediated inflammation and vascular damage. Importantly, AngII markedly increased aortic expression of inflammatory genes including TNF-α, MCP-1, IL-6, IL-12, ICAM-1, CCR2 and osteopontin in HF/AngII/AdAPN mice compared to HF/PBS mice (Figure 5). APN significantly suppressed the expression of all of these AngII-induced inflammatory genes in the artery wall (Figure 5). In case of the anti-inflammatory cytokine, IL-10, we found that AngII substantially suppressed its expression whereas APN expression significantly increased IL-10 in the artery wall (Figure 5). Taken together, these data demonstrate that APN potently inhibits AngII-induced inflammation and induces anti-inflammatory factors in the artery wall.

### Adiponectin Expression Inhibits Expression of Scavenger Receptors and Increases Cholesterol Efflux Transporters in the Artery Wall

**Figure 6:**
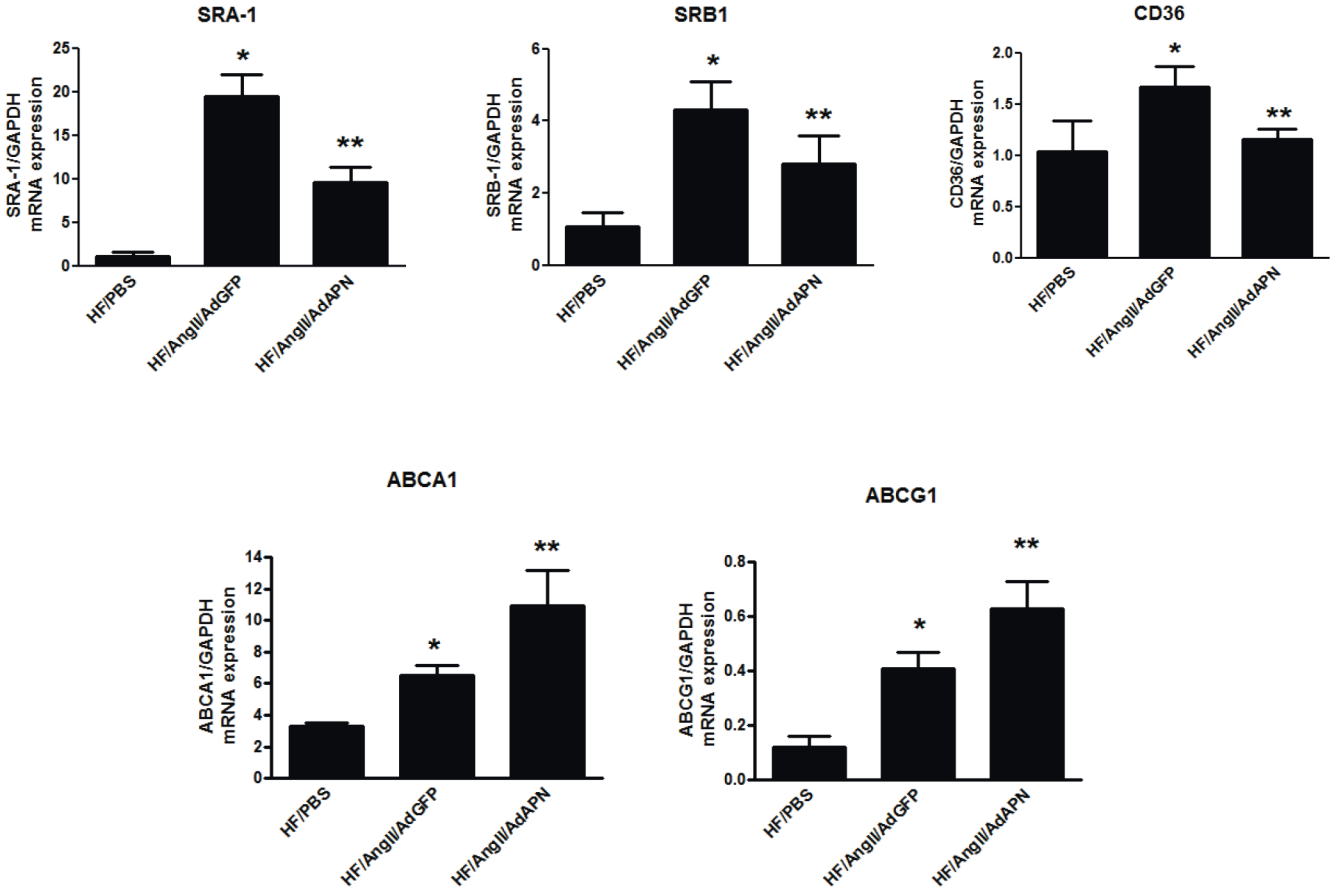
Adiponectin expression inhibits scavenger receptor gene expression and increases cholesterol efflux receptors in the artery wall. (A) RNA isolated from aorta samples were analyzed for scavenger receptors SR-A1, SR-B1 and CD36 and (B) cholestrol efflux receptors, ABCA1 and ABCG1 expression by QRT-PCR and normalized to GAPDH expression (*p<0.05 vs HF, **p<0.05 vs HF/AngII/AdGFP Newman-Keuls, n = 5–7/group).

AngII is a strong inducer of foam cell formation and suppresses the expression of the cholesterol efflux transporter ABCA1, decreasing macrophage cholesterol efflux and accelerating atherosclerotic lesion development [44]. Since cholesterol homeostasis is critical in atherosclerosis, we examined the expression of both scavenger receptors and cholesterol efflux promoting ABC transporters in the artery wall. Importantly, aortic expression of cholesterol uptake genes (SR-A1, SR-B1 and CD36) was substantially increased in HF/AngII/AdGFP mice compared with control HF/PBS mice (Figure 6). Interestingly, APN expression significantly suppressed the aortic mRNA levels of SR-A1, SR-B1 and CD36 (Figure 6). These data indicate that APN inhibits AngII-induced scavenger receptor expression to prevent aortic cholesterol deposition and foam cell formation. On the other hand, analysis of aortas from HF/AngII/AdAPN 8-week treated mice revealed a significant increase in the expression of ABCA1 and ABCG1 compared to HF/AngII/GFP mice despite a significant reduction in lesion macrophage content (Figure 6). These results provide important evidence that APN limits cholesterol deposition and promotes cholesterol efflux from the artery wall thereby decreasing foam cell formation and atherosclerosis.

## Discussion

**Figure 7:**
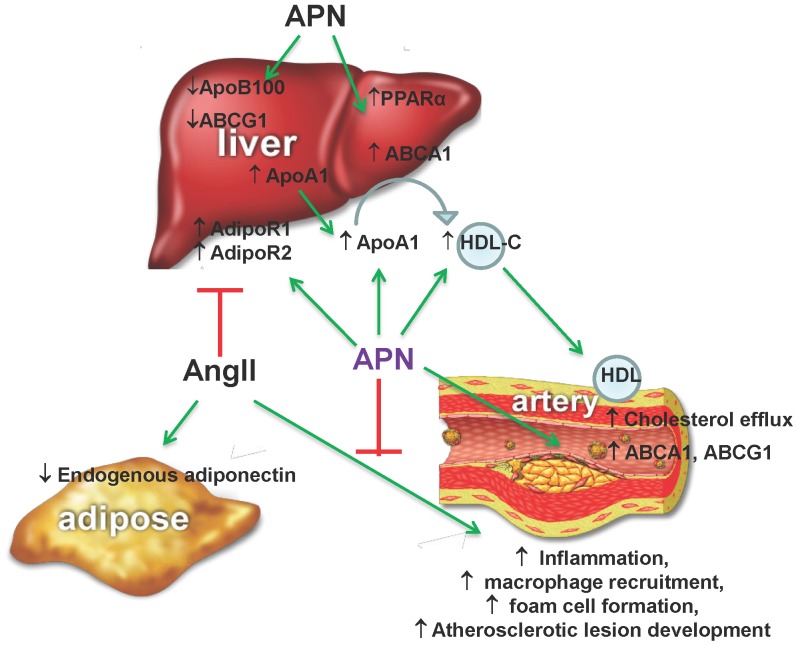
Schematic diagram showing atheroprotective mechanisms of APN. Results from this study demonstrated that APN inhibits AngII effects on hepatic adiponectin receptors (AdipoR1 and AdipoR2) and metabolic gene expression (ApoA1, ABCA1), plasma HDL levels, vascular inflammation and atherosclerotic lesion development.

The pathogenesis of atherosclerosis involves increased vascular inflammation and metabolic dysregulation. Available evidence strongly supports a role for APN as a potent modulator of both inflammation and cholesterol metabolism and thereby as an emerging therapeutic target for atheroprotection [6], [12], [30]. However, prior studies on the effects of APN in preclinical models of atherosclerosis are limited and have produced diverse results [36], [37]. Thus, we addressed the hypothesis that elevation of plasma APN levels inhibits AngII-mediated vascular inflammation and protects against metabolic syndrome-associated atherosclerosis. Using a well-established, AngII-infused hyperlipidemic LDLR^−/−^ mouse, we demonstrated that APN expression provides strong atheroprotection without blood pressure reduction. Adenoviral APN expression increased plasma APN levels higher than GFP-vector and PBS-injected mice for up to 8 weeks as in previous reports using second/third generation adenoviral vectors expressing plasma apolipoproteins in atherosclerosis intervention studies [45], [46]. APN expression increased circulating HMW APN levels leading to elevated plasma HDL and ApoA1 levels as well as improved metabolic gene expression profiles in the liver. Importantly, we demonstrated that increasing plasma APN levels modulate AngII receptor expression and inhibit AngII-mediated inflammatory and atherogenic gene expression in the artery wall (Figure 7).

Previously we have shown that AngII profoundly increases vascular inflammation and atherosclerosis in high-fat fed LDLR^−/−^mice [38]. Here, we showed that AngII infusion for 8 weeks significantly suppressed endogenous APN expression in the white adipose tissue. Adenoviral APN expression had no effect on the AngII-induced suppression of adipose APN expression. Importantly, in the AngII-infused mice, adenoviral APN expression resulted in a substantial increase in the plasma levels of both total and HMW APN. Several studies have shown that HMW APN is a better biomarker than total APN and is also more active against insulin resistance and metabolic syndrome [10], [47]. A number of clinical studies reported a direct and positive correlation between plasma HMW APN and HDL-C levels [14], [15]. Consistent with these data, in our study the elevation of APN as well as HMW APN also led to increased plasma HDL-C levels in our hypertensive and hyperlipidemic model of atherosclerosis.

The liver is the major organ controlling lipid metabolism, HDL biogenesis and reverse cholesterol transport [41], [42]. We demonstrated that APN induces the hepatic expression of APN receptors and genes regulating HDL and lipid homeostasis. Previous *in vitro* studies reported that APN increases ApoA1 and ABCA1 expression in hepatocytes contributing to HDL formation [43]. Furthermore, both ABCA1 and ApoA1 expression in the liver are reduced in APN-deficient mice [48]. Our studies provided *in vivo* evidence that APN expression increased ApoA1 protein levels in the liver and plasma, accompanied by HDL-C elevation. Recent evidence suggests that hepatic ABCA1 expression is a major regulator of plasma HDL-C levels contributing up to 80% to total plasma HDL-C levels [49]. In the current study, we found that APN expression significantly increased ABCA1 mRNA levels in the liver. In the context of hepatic HDL metabolism, PPARα, a nuclear hormone receptor, plays a key role in the regulation of HDL and triglyceride metabolism. In this study, APN prevented AngII-mediated downregulation of the APN receptors, AdipoR1 and AdipoR2 in the liver. APN signaling through AdioR2 has been shown to activate PPARα [13], [16]. Consistent with the APN induction of AdipoR2 in the liver, we also showed that APN increased expression of PPARα in AngII-infused mice. Although lipoprotein profiles of mice expressing APN revealed only modest changes in cholesterol within ApoB-containing lipoproteins, we found a suppression of hepatic ApoB100. Short-term adenoviral APN expression has been previously reported to reduce plasma triglyceride levels and hepatic ApoB mRNA levels [50]. In addition, *in vitro* studies with human hepatocytes showed that HMW APN reduced ApoB secretion [51]. Our data demonstrated that APN altered hepatic gene expression, increasing plasma HDL-C and ApoA1 levels while reducing the expression of ApoB100. Importantly, these favorable changes in gene expression profile together with an elevation of HDL-C can contribute to atheroprotection by APN. In contrast, AngII induced the expression of ABCG1, which has been reported to be pro-atherogenic [52], while APN significantly reduced its expression. Thus, our results suggest that APN exerts several beneficial actions in the liver that can contribute to atheroprotection.

A number of clinical and preclinical studies have demonstrated that elevated plasma APN levels are associated with a low-incidence of cardiovascular disease [12]. Deficiency of APN in ApoE^−/−^ mice promotes atherosclerosis and T-lymphocyte accumulation in the atherosclerotic lesions [36], [53]. Replenishment of APN by adenoviral expression in these mice attenuated atherosclerotic lesion formation [36]. In contrast, other studies have reported that neither genetic overexpression nor APN knockout had any significant effect on atherosclerosis in low-fat or high-fat fed LDLR^−/−^ mice or ApoE^−/−^ mice [37]. The reasons for the differential APN effects observed in previous studies remains unclear. It is possible that differing APN expression levels, metabolic and inflammatory factors in previous dietary/genetic models vs AngII-infused hypertensive model may have contributed to the divergent outcomes. Here, in the hypertensive, hyperlipidemic LDLR^−/−^ mouse model of accelerated atherosclerosis, APN expression effectively suppressed macrophage recruitment and vascular inflammation, contributing to a substantial inhibition of AngII-accelerated atherosclerosis. It is noteworthy that APN expression did not affect blood pressure, suggesting that APN exerts direct anti-inflammatory and anti-atherogenic actions on the vascular wall independent of blood pressure reduction.

There is evidence suggesting that circulating APN enters the arterial wall and binds to collagens in the sub-endothelial space of damaged arteries [54]. In this study, immunohistochemical analysis not only demonstrated the presence of APN in aortic lesions of AngII-infused mice but also indicated that the APN localization in lesions was markedly enhanced in APN expressing mice. AngII-infusion induces endothelial dysfunction due to enhanced macrophage recruitment, inflammation and oxidative stress causing vascular damage [17], [18], [19]. This study showed that elevation of APN levels significantly inhibited macrophage recruitment and inflammation in the artery wall.

APN signaling pathways downstream of AdipoR1 and AdipoR2 include activation of AMPK and PPARα leading to transrepression of NF-κB and repression of inflammatory gene expression [55], [56], [57], [58], [59], [60]. In addition, APN mediated ceramide signaling through depletion of ceramide, independently of AMPK and PPARα, can also contribute to anti-inflammatory effects [61]. We demonstrated that elevation of APN levels suppressed aortic expression of key inflammatory genes (TNF-α, IL-6, IL-12) in a hypertensive, pro-inflammatory and hyperlipidemic model of atherosclerosis [38]. Furthermore, we have shown a significant inhibition of MCP-1 and its receptor CCR2, the chemokine pathway induced by AngII that contributes to macrophage recruitment and acceleration of atherosclerosis. Previous studies reported APN suppression of nuclear factor κB (NFkB)-inducible genes [36]. The AngII actions contributing to inflammation and vascular damage are mediated by the angiotensin II type-1 receptor (AT1R) expressed on all vascular cells. Here, we showed that APN expression inhibited AngII-induced expression of AT1R in the artery wall. In contrast, AngII suppressed the aortic expression of angiotensin II type-2 receptor (AT2R), which antagonizes AngII signaling and contributes to ant-inflammatory effects [62]. Interestingly, APN expression increased the aortic expression of AT2R. These findings indicate that APN regulates the expression of AngII receptors which can potentially modulate vascular actions of AngII in the artery wall.

Clearly, results from this study support the beneficial role of APN in the suppression of RAS-mediated vascular inflammation and macrophage accumulation leading to atheroprotection. In addition, we also demonstrated that APN increased the expression of the anti-inflammatory cytokine, IL-10 in the artery wall. Recent evidence implicated APN as an important regulator of macrophage polarization. Consistent with its anti-inflammatory properties, APN has been shown to promote alternative (M2) activation of macrophages [63], whereas pro-inflammatory conditions lead to the classical (M1) activation. Interestingly, in AngII-infused mice we have shown that APN inhibited aortic IL-12 and increased IL-10, markers of M1 and M2, respectively. This suggests the possibility that APN may affect macrophage properties, including their polarization, in atherosclerotic lesions.

Previous studies have shown that AngII promotes macrophage foam cell formation, which is critical in the pathogenesis of atherosclerosis [44]. Foam cell formation is regulated by the net balance between cholesterol influx, mediated by the scavenger receptors, and cholesterol efflux, facilitated by the ATP-binding cassette transporters [41]. Furthermore, AngII induces scavenger receptors, CD36 and SR-A1 in macrophages promoting the uptake of modified lipoproteins and cholesterol accumulation. It has been reported that adenoviral APN expression for 2 weeks reduced the expression of scavenger receptor A1 (SR-A1) but had no effect on CD36 in the aorta in ApoE^−/−^ mice [36]. In this study, adenoviral expression of APN for 8 weeks in AngII-accelerated LDLR^−/−^ atherosclerotic mice significantly reduced the aortic expression of SR-A1, CD36 and SR-B1. Thus, APN suppresses scavenger receptor expression to reduce macrophage uptake of modified lipoproteins and foam cell development in AngII accelerated atherosclerosis. As shown previously, in hypertensive, hyperlipidemic LDLR^−/−^ mice, ABCA1 and ABCG1 are predominantly expressed by macrophages in the artery wall [44]. Our data indicate that APN expression substantially increases the expression of cholesterol efflux transporters, ABCA1 and ABCG1 in the artery wall. APN-mediated activation of PPARα induces LXR target genes, ABCA1 and ABCG1 [55]. Thus, APN-mediated increase in the artery wall expression of these efflux receptors suggests that elevated APN levels effectively promote reverse cholesterol transport. Taken together, these results suggest that APN inhibits macrophage foam cell development by suppressing scavenger receptors and increasing efflux transporters to inhibit AngII-accelerated atherosclerosis.

This study provides important evidence that APN expression exerts profound anti-inflammatory, metabolic and anti-atherogenic actions both in the liver and the artery wall to effectively inhibit AngII-mediated vascular inflammation and accelerated atherosclerosis (Figure 7). Remarkably, the atheroprotective actions of APN are independent of blood pressure reduction. These studies strongly support that concept that increasing plasma APN levels may be an effective therapeutic strategy to inhibit increased vascular inflammation and accelerated atherosclerosis, especially relevant to cardiovascular complications triggered by RAS activation in the metabolic syndrome.
